# Protective effect of the Quercetin on experimental cuprizone-induced multiple sclerosis in male C57BL/6 mice

**DOI:** 10.1016/j.crphys.2025.100146

**Published:** 2025-05-23

**Authors:** Samin Ghasemi, Shahin Hassanpour, Razieh Hosseini

**Affiliations:** a Faculty of Veterinary Medicine, SR.C., Islamic Azad University, Tehran, Iran; b Department of Basic Veterinary Sciences, SR.C., Islamic Azad University, Tehran, Iran

**Keywords:** Quercetin, Cuprizone, Multiple sclerosis, Mice

## Abstract

This study aimed to determine protective effect of the Quercetin on experimental cuprizone-induced (CPZ) multiple sclerosis (MS) in Male C57BL/6 mice. Forty male C57BL/6 mice were allocated into 4 experimental groups. Control group received the standard diet. In group 2, mice received a diet containing 0.2 % (w/w) CPZ mixed with ground chow for a duration of 4 weeks (Zhu et al., 2021). In group 3, mice received Quercetin (150 mg/kg) orally every day for 4 weeks. In group 4, mice received a diet with 0.2 % CPZ and Quercetin (150 mg/kg) orally for 4 weeks. After incidence of the sign of the MS reflexive motor behavior and serum antioxidant levels were assessed. Based on the findings, administration of the CPZ significantly decreased ambulation score, number of crosses using OFT (open field test), stay on rotarod, hindlimb foot angle, front- and hindlimb suspension and grip strength (P < 0.05). Quercetin significantly increased ambulation score, number of cross using, stay on rotarod, hindlimb foot angle, front- and hindlimb suspension and grip strength (P < 0.05). Administration of the CPZ significantly increased serum malondialdehyde (MDA), tumor necrosis factor (TNF-α) and interleukin-1 beta (IL-1β) While decreased superoxide dismutase (SOD), glutathione peroxidase (GPX) and total antioxidant status (TAS) levels (P < 0.05). Quercetin significantly decreased serum MDA, TNF-α and IL-1β while increased SOD, GPX and TAS levels (P < 0.05). These results suggested, Quercetin has protective effect against CPZ-induced MS in mice.

## Highlights

• CPZ significantly increased serum MDA, TNF-α and IL-1β While decreased SOD, GPX and TAS levels.
•Quercetin significantly decreased serum MDA, TNF-α and IL-1β while increased SOD, GPX and TAS levels.
•Quercetin has protective effect against CPZ-induced MS in mice.

## 1 Introduction

Flavonoids, known as bioflavonoids, are identified as secondary compounds found in fungi and plants containing a 15-carbon structure, two phenyl rings, and a heterocyclic ring (Li et al., 2022). More than 5000 flavonoids are found in various herbs. Quercetin, identified as 3,5,7,3′,4′-pentahydroxyflavone, is present in many types of flowers, roots, vegetables, and fruits. It also demonstrates a variety of intriguing treatments for different health issues such as anti-cancer, anti-inflammatory, anti-diabetes, and antioxidant effects (Hosseini et al., 2021). Quercetin is primarily known for its antioxidant capabilities (Di Petrillo et al., 2022). It defends our body against free radicals and is the most potent antioxidant flavonoid found in nature (Di Petrillo et al., 2022). Chen and colleagues (2021) demonstrated that Quercetin has a beneficial effect on diabetes control and improves lipid profile through its antioxidant and nitric oxide properties.

It is widely agreed that MS is an autoimmune disorder that affects the central nervous system (CNS) by attacking the myelin. Despite the initial medical descriptions of MS in the early 18th century, little advancement in comprehending MS pathology occurred in the 18th and 19th century due to research being restricted to post-mortem analysis of brain tissue from MS patients (Zhang et al., 2020). Throughout the 20th century, various animal models for MS that are immune-mediated were created, such as the experimental autoimmune encephalomyelitis (EAE) model and the Theiler murine encephalomyelitis virus model. In addition to these, other models induced by toxins such as the cuprizone (CPZ) model, the lysophosphatidyl choline injection model, and the ethidium bromide injection model are commonly utilized to study the molecular aspects of demyelination and remyelination (Avşar et al., 2021).

Although the CPZ mouse model has been used on various animal species and mouse strains over the last 40 years, it is now primarily utilized on mouse C57BL/6 background because it consistently shows de- and remyelination along with microgliosis and astrogliosis (Nicola et al., 2024). Furthermore, the presence of numerous transgenic mouse strains bred on a C57BL/6 background, along with the consistent success of the CPZ model on this background, positions CPZ intoxication as an advantageous model for examining acute and chronic demyelination, as well as remyelination (Shao et al., 2021).

Lymphocytes that cross the blood brain barrier and get activated are the cause of the CNS lesions in MS. The continuous neuroinflammation from the immune response leads to oxidative stress, cytotoxicity, axonal damage, astrogliosis, and microgliosis. Current treatment strategies for multiple sclerosis aim to decrease the immune response, while also working towards addressing the root cause of the disease or supporting the repair of damaged tissues (Filippi et al., 2022). Based on the literature Quercetin modulates immune response in peripheral blood mononuclear cells isolated from multiple sclerosis patients (Sternberg et al., 2008). Also, Quercetin attenuates neuroinflammation via its antioxidant activity (Bahar et al., 2017). Quercetin exerts neuroprotective action through inhibition of inducible nitric oxide synthase (Kang et al., 2013). Still, these treatments only provide partial relief for MS and mainly have varying impacts on the degenerative aspects of the condition, along with expensive costs and unfavorable safety records. As a result, there is an ongoing requirement for new drugs that possess enhanced safety profiles and innovative modes of action (Coles et al., 2022). Thus, this study aimed to determine protective effect of the Quercetin on experimental CPZ-induced MS in Male C57BL/6 mice.

## 2 Material and methods

### 2.1 Animals

Forty male C57BL/6 mice (aged 4–6 weeks and weighing 20 ± 2 g) were kept in controlled conditions (temperature: 22 °C ± 2 °C, 12-h light/dark cycle). They were able to obtain regular food and water. Following a week of adjustment, the mice were divided into four groups randomly (n = 10). The research committee of Islamic Azad University, Science and Research Branch approved the study protocols. Then animals were allocated into 4 experimental groups. Control group received the standard diet. In group 2, mice received a diet containing 0.2 % (w/w) CPZ (Sigma Aldrich, St. Louis, MO, USA) mixed with ground chow for a duration of 4 weeks (Zhu et al., 2021). In group 3, mice received Quercetin (Sigma Aldrich, St. Louis, MO, USA, 150 mg/kg) orally every day for 4 weeks. In group 4, mice received a diet with 0.2 % CPZ and Quercetin (150 mg/kg) orally for 4 weeks. During this time, mice in groups 2–4 were injected once daily with 10 mg/kg rapamycin intraperitoneally. The experimental autoimmune encephalomyelitis (EAE) scoring system was used to quantify disease severity on a scale of 0–5, as follows: 0: no disease, 1: loss of tail tone, 2: hind limb weakness, 3: hind limb paralysis, 4: hind limb and forelimb paralysis or weakness, and 5: moribund/death (Graves and Montalban, 2020). Then, reflexive motor behavior and serum antioxidant levels were assessed.

### 2.2 Behavioral tests

#### 2.2.1 Ambulation

The mice were put in an opaque container that was visible from all angles during the test, and they were encouraged to move by mildly stimulating their tails. A scoring system was used to evaluate their ambulation, with 0 indicating no movement, 1 for crawling with asymmetric limb movement, 2 for slow crawling with symmetric limb movement, and 3 for fast crawling or walking. In order to mitigate the effect of learning, the test was repeated three times within a 3-min window (Feather-Schussler and Ferguson, 2016).

#### 2.2.2 Open field test

This test evaluated locomotor and exploratory activities. Mice were placed in a divided square wooden box (45 × 45 × 30 cm3 with nine divided squares) and the number of squares crossed was recorded in 6 min.

#### 2.2.3 Rotarod test

This test assessed motor coordination and balance. Mice were placed on a rotating rod, and the time until falling off was recorded.

#### 2.2.4 Hind limb foot angle

Mouse movements were recorded in an open field box using a camera. Images capturing hind limb positions during full stride were used. A line was drawn from the heel to the tip of the toe, and the angle between them was measured (Feather-Schussler and Ferguson, 2016).

#### 2.2.5 Hind limb suspension

Mice's hind legs were hung over a wire tied between two ends, and their posture was observed. Hind limb posture was scored based on the time to grasp the wire and the separation of hind limbs (Feather-Schussler and Ferguson, 2016).

#### 2.2.6 Grip strength

Mice were placed on a fiberglass screen and slowly tilted from horizontal to vertical. Mice attempted to grip the screen to prevent falling. The time until falling was recorded (Corti, 2017).

#### 2.2.7 Negative geotaxis

Mice were placed face down on a 45° incline. The time taken to turn and face upwards was recorded (Feather-Schussler and Ferguson, 2016).

#### 2.2.8 Front limb suspension

This test evaluated the ability of animals to hang by their front limbs. Briefly, mice grasped a wire suspended between two ends. The time until mice released the wire was recorded. This test was repeated three times, and the average time in seconds was recorded (Feather-Schussler and Ferguson, 2016).

#### 2.2.9 Antioxidant activity

Blood samples were taken to measure serum MDA, SOD, GPx, TAS, TNF-α and IL-1β activities using Randox (UK, England) ELISA assay kits (Bradford, 1976).

#### 2.2.10 Statistical analysis

Data were analyzed using one-way ANOVA and were presented as mean ± SE (standard error) using SPSS version 22.0. For treatments showing significant differences by ANOVA, between-group evaluations were performed using the Tukey posthoc test (p < 0.05).

## 3 Results

**Fig. 1:**
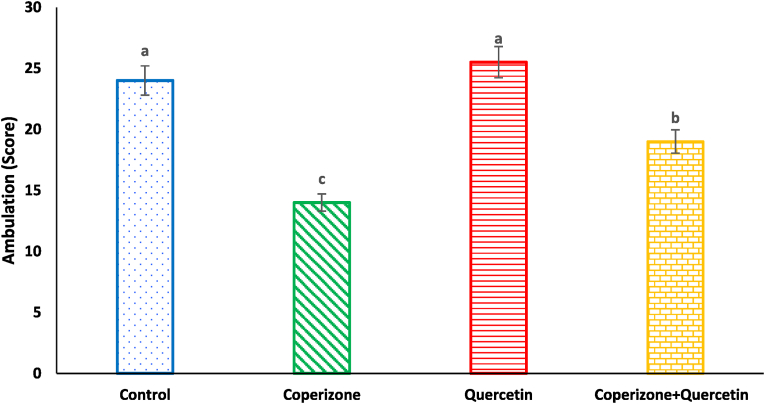
Effects of the Quercetin on ambulation score in experimental cuprizone-induced Multiple Sclerosis Male C57BL/6 mice. Data are expressed as mean ± SE. There are significant differences between groups with different superscripts (a-c; P < 0.05).

The administration of the CPZ significantly decreased ambulation score compared to the control group (P < 0.05). Quercetin had no significant effect on ambulation score compared to the control group (P < 0.05). Co-administration of the Quercetin and CPZ significantly increased ambulation score compared to the CPZ group (P < 0.05) (Fig. 1).

**Fig. 2:**
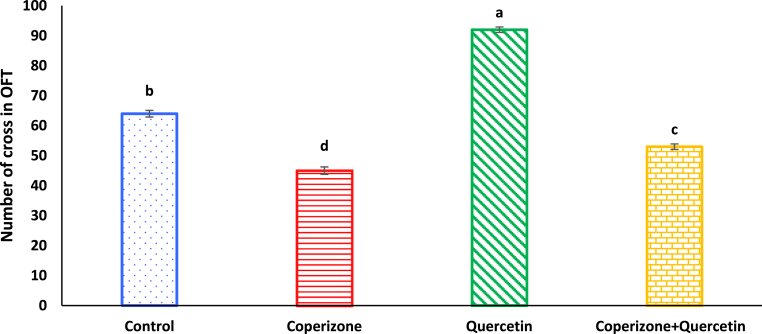
Effects of the Quercetin on number of cross using OFT in experimental cuprizone-induced Multiple Sclerosis Male C57BL/6 mice. Data are expressed as mean ± SE. There are significant differences between groups with different superscripts (a-d; P < 0.05).

Based on the findings, administration of the CPZ significantly had adverse effect on number of crosses using OFT compared to control group (P < 0.05). Quercetin significantly increased number of crosses using OFT in comparison compared to control group (P < 0.05). Co-administration of the Quercetin and CPZ significantly increased number of cross compared to the CPZ group (P < 0.05) (Fig. 2).

**Fig. 3:**
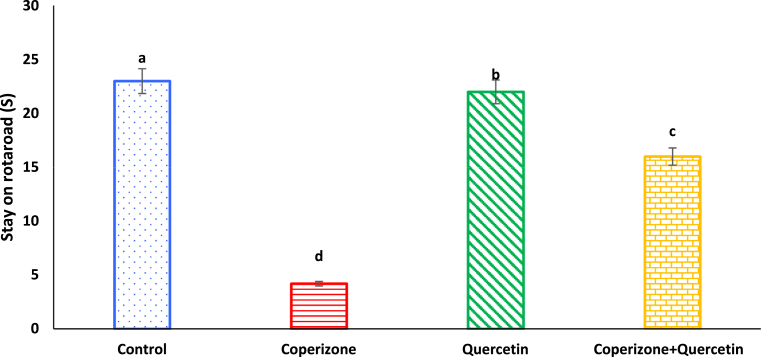
Effects of the Quercetin on stay on rotarod in experimental cuprizone-induced Multiple Sclerosis Male C57BL/6 mice. Data are expressed as mean ± SE. There are significant differences between groups with different superscripts (a-d; P < 0.05).

As seen, administration of the CPZ significantly decreased stay on rotarod compared to control group (P < 0.05). Quercetin significantly increased stay on rotarod than to control group (P < 0.05). Co-administration of the Quercetin and CPZ significantly increased stay on rotarod compared to the CPZ group (P < 0.05) (Fig. 3).

**Fig. 4:**
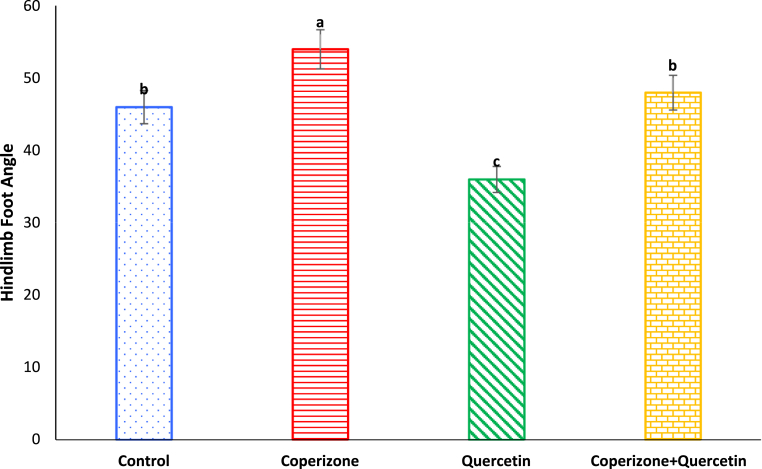
Effects of the Quercetin on hindlimb foot angle in experimental cuprizone-induced Multiple Sclerosis Male C57BL/6 mice. Data are expressed as mean ± SE. There are significant differences between groups with different superscripts (a-c; P < 0.05).

CPZ significantly decreased hindlimb foot angle compared to control group (P < 0.05). Hindlimb foot angle significantly increased following Quercetin administration compared to control group (P < 0.05). Co-administration of the Quercetin and CPZ significantly decreased hindlimb foot angle compared to the CPZ group (P < 0.05) (Fig. 4).

**Fig. 5:**
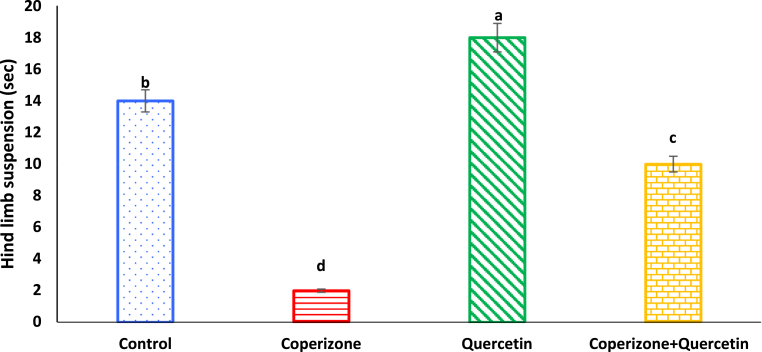
Effects of the Quercetin on hindlimb suspension in experimental cuprizone-induced Multiple Sclerosis Male C57BL/6 mice. Data are expressed as mean ± SE. There are significant differences between groups with different superscripts (a-d; P < 0.05).

As seen in Fig. 5, hindlimb suspension significantly decreased by CPZ administration compared to control group (P < 0.05). Quercetin administration significantly increased hindlimb suspension compared to control group (P < 0.05). Co-administration of the Quercetin and CPZ significantly increased hindlimb suspension compared to the CPZ group (P < 0.05).

**Fig. 6:**
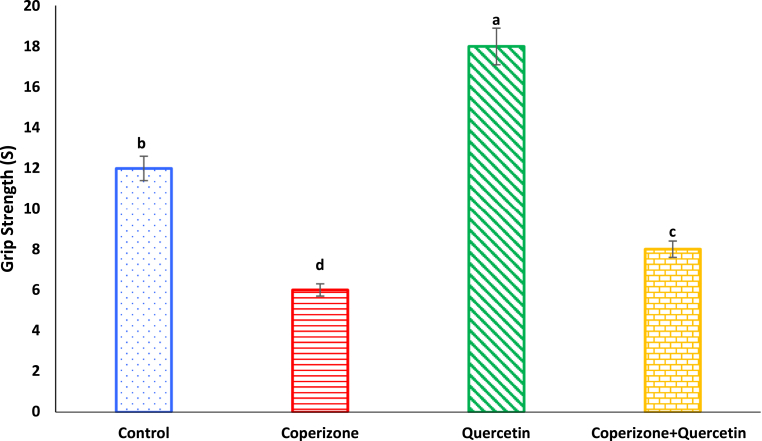
Effects of the Quercetin on grip strength in experimental cuprizone-induced Multiple Sclerosis Male C57BL/6 mice. Data are expressed as mean ± SE. There are significant differences between groups with different superscripts (a-d; P < 0.05).

In this study, grip strength significantly decreased by CPZ administration compared to control group (P < 0.05). Quercetin administration significantly increased grip strength compared to control group (P < 0.05). Grip strength increased by co-administration of the Quercetin and CPZ compared to the CPZ group (P < 0.05) (Fig. 6).

**Fig. 7:**
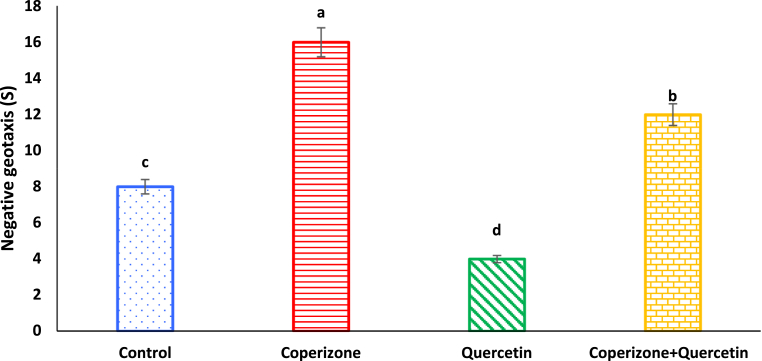
Effects of the Quercetin on negative geotaxis in experimental cuprizone-induced Multiple Sclerosis Male C57BL/6 mice. Data are expressed as mean ± SE. There are significant differences between groups with different superscripts (a-d; P < 0.05).

As shown, negative geotaxis significantly decreased by CPZ administration compared to control group (P < 0.05). Quercetin administration significantly increased negative geotaxis compared to control group (P < 0.05). Negative geotaxis increased by co-administration of the Quercetin and CPZ compared to the CPZ group (P < 0.05) (Fig. 7).

**Fig. 8:**
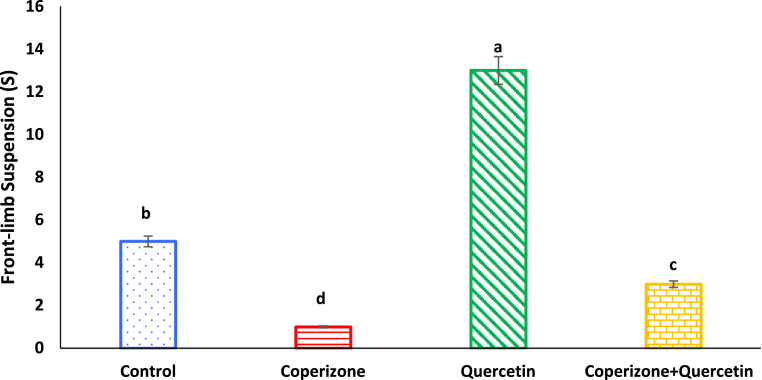
Effects of the Quercetin on front-limb suspension in experimental cuprizone-induced Multiple Sclerosis Male C57BL/6 mice. Data are expressed as mean ± SE. There are significant differences between groups with different superscripts (a-d; P < 0.05).

Based on our findings, front-limb suspension significantly decreased in mice received CPZ in comparison to control group (P < 0.05). Quercetin administration significantly increased front-limb compared to control group (P < 0.05). Co-administration of the Quercetin and CPZ significantly increased front-limb suspension compared to the CPZ group (P < 0.05) (Fig. 8).

**Fig. 9:**
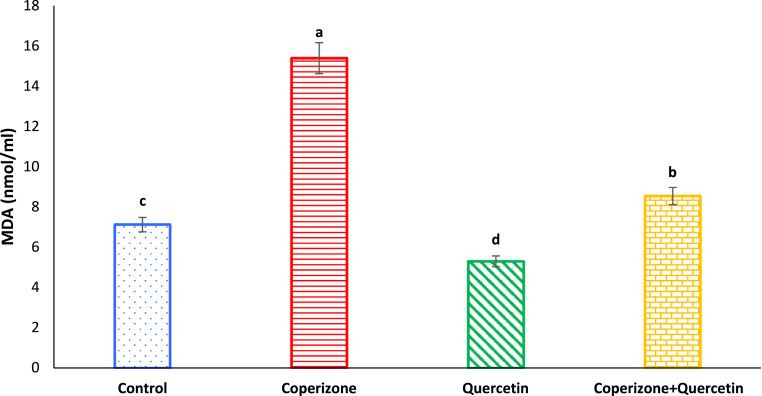
Effects of the Quercetin on serum MDA in experimental cuprizone-induced Multiple Sclerosis Male C57BL/6 mice. Data are expressed as mean ± SE. There are significant differences between groups with different superscripts (a-d; P < 0.05).

As seen in Fig. 9, serum MDA levels significantly increased in CPZ treated mice in comparison to control group (P < 0.05). Quercetin administration significantly decreased serum MDA compared to control group (P < 0.05). Co-administration of the Quercetin and CPZ significantly decreased serum MDA compared to the CPZ group (P < 0.05).

**Fig. 10:**
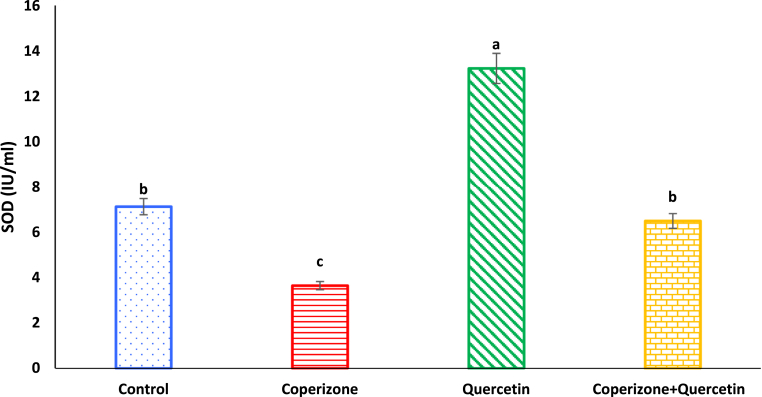
Effects of the Quercetin on serum SOD in experimental cuprizone-induced Multiple Sclerosis Male C57BL/6 mice. Data are expressed as mean ± SE. There are significant differences between groups with different superscripts (a-d; P < 0.05).

As observed, serum SOD levels significantly decreased in CPZ treated mice in comparison to control group (P < 0.05). Quercetin administration significantly increased serum SOD compared to control group (P < 0.05). Co-administration of the Quercetin and CPZ significantly increased serum SOD compared to the CPZ group (P < 0.05) (Fig. 10).

**Fig. 11:**
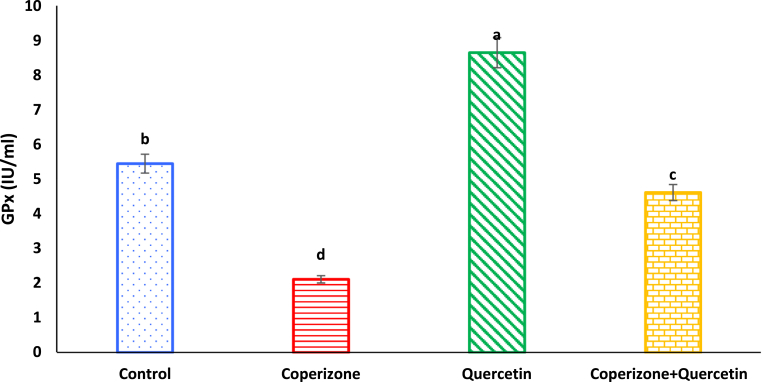
Effects of the Quercetin on serum GPx in experimental cuprizone-induced Multiple Sclerosis Male C57BL/6 mice. Data are expressed as mean ± SE. There are significant differences between groups with different superscripts (a-d; P < 0.05).

Based on Fig. 11, serum GPx levels significantly decreased in CPZ treated mice in comparison to control group (P < 0.05). Quercetin administration significantly increased serum GPx compared to control group (P < 0.05). Co-administration of the Quercetin and CPZ significantly increased serum GPx compared to the CPZ group (P < 0.05).

**Fig. 12:**
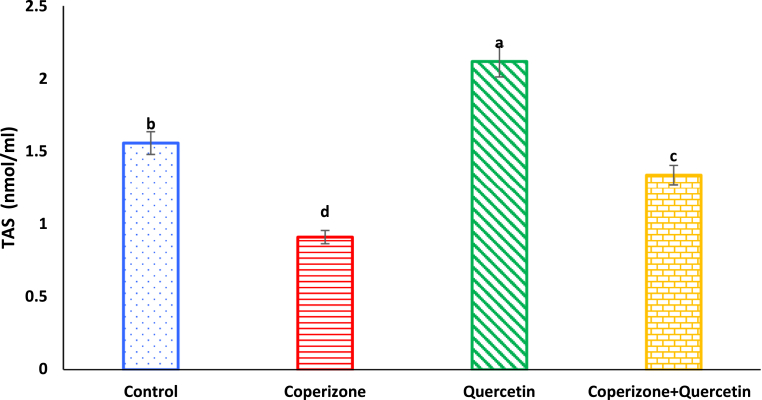
Effects of the Quercetin on serum TAS in experimental cuprizone-induced Multiple Sclerosis Male C57BL/6 mice. Data are expressed as mean ± SE. There are significant differences between groups with different superscripts (a-d; P < 0.05).

In our results, serum TAS levels significantly decreased in CPZ treated mice in comparison to control group (P < 0.05). Quercetin administration significantly increased serum TAS compared to control group (P < 0.05). Co-administration of the Quercetin and CPZ significantly increased serum TAS compared to the CPZ group (P < 0.05) (Fig. 12).

**Fig. 13:**
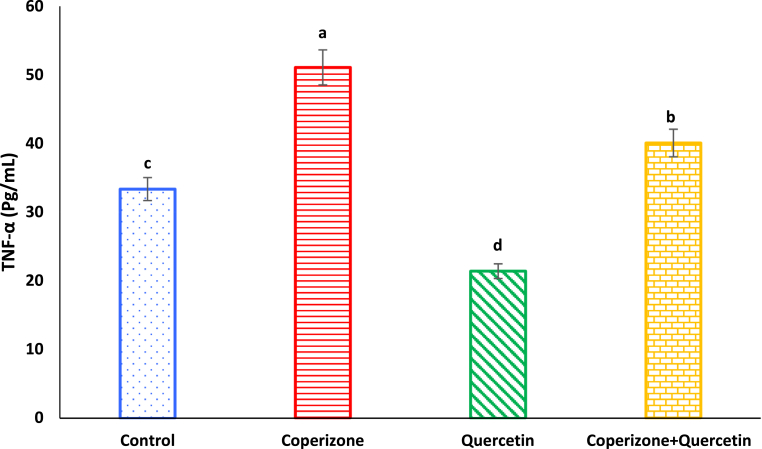
Effects of the Quercetin on serum TNF-α in experimental cuprizone-induced Multiple Sclerosis Male C57BL/6 mice. Data are expressed as mean ± SE. There are significant differences between groups with different superscripts (a-d; P < 0.05).

As seen, CPZ administration, significantly increased serum TNF-α compared to control group (P < 0.05). Quercetin administration significantly decreased serum TNF-α compared to control group (P < 0.05). Co-administration of the Quercetin and CPZ significantly decreased serum TNF-α compared to the CPZ group (P < 0.05) (Fig. 13).

**Fig. 14:**
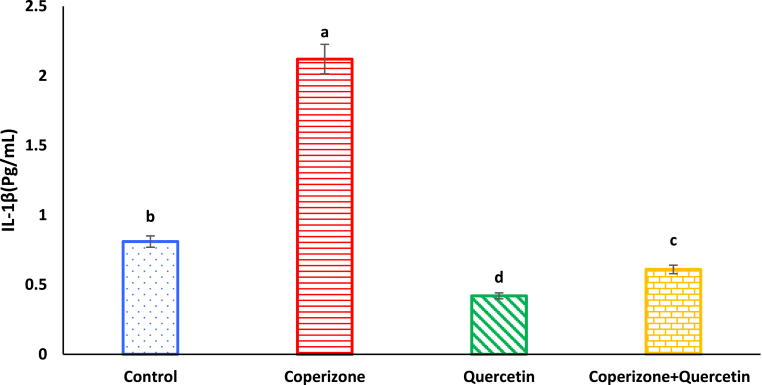
Effects of the Quercetin on serum IL-1β in experimental cuprizone-induced Multiple Sclerosis Male C57BL/6 mice. Data are expressed as mean ± SE. There are significant differences between groups with different superscripts (a-d; P < 0.05).

According to Fig. 14, CPZ administration, significantly increased serum IL-1β compared to control group (P < 0.05). Quercetin administration significantly decreased serum IL-1β compared to control group (P < 0.05). Co-administration of the Quercetin and CPZ significantly decreased serum IL-1β compared to the CPZ group (P < 0.05).

## 4 Discussion

Based on the findings, administration of the CPZ decreased ambulation score, number of crosses using OFT, stay on rotarod, hindlimb foot angle, front- and hindlimb suspension and grip strength. Administration of the CPZ significantly increased serum MDA, TNF-α and IL-1β While decreased SOD, GPX and TAS levels. Mice exposed to 0.2 % CPZ for 4–6 weeks had impaired sensorimotor gating and less social interaction, resulting in staying in the open arms of the maze and more memory impairment (Xu et al., 2009; Ojha, 2018). One of the aspects of the MS that is pathological is the excess oxidative stress levels generated by the immune cells in the central nervous system of individuals with the condition. It has been shown that oxidative stress is the primary cause of demyelination in the CPZ-induced model (Tomas-Roig et al., 2020). Several research studies investigated the detrimental effects of CPZ on oligodendrocytes. Research has demonstrated that after 5 weeks of CPZ intoxication, the activity of Cu/Zn SOD significantly reduces, leading to a lack of the enzyme in removing ROS (Tahmasebi et al., 2021). The build-up of ROS in oligodendrocytes results in activation of pathways that harm the cells such as increased lipid peroxidation, reduced GSH levels, lowered ferritin levels, and ultimately cell demise (Sen et al., 2020). Most studies have shown that inflammation is a central factor in the development of MS (Zahednasab et al., 2019). The overactive inflammatory responses can worsen the progression of the disease by creating an abundance of proinflammatory cytokines in both the central nervous system and the rest of the body. Researchers are looking for ways to regulate the levels of inflammatory cytokines due to the imbalanced profile of proinflammatory mediators (Nicola et al., 2024). Current data shows that CPZ increases TNF-α and IL-1β levels in mouse serum which was in agreement to previous reports (Jiménez-Jiménez et al., 2024).

As seen from our findings, Quercetin increased number of cross using, stay on rotarod, hindlimb foot angle, front- and hindlimb suspension and grip strength. Quercetin decreased serum MDA, TNF-α and IL-1β while increased SOD, GPX and TAS levels. The documented neuroprotective benefits of flavonoids against different neurodegenerative conditions are well established (Yu et al., 2020). Flavonoids possess various advantageous medicinal qualities such as anti-inflammatory, antioxidant, anti-carcinogenic, and anti-mutagenic effects (Behrangi et al., 2017). Numerous research efforts have been carried out to assess how flavonoids impact antioxidant properties in conditions like Alzheimer's and Parkinson's disease. Lately, there has been a lot of focus on researching the impact of polyphenols on MS disease because of their abilities to modulate the immune system, reduce inflammation, and act as antioxidants (Omotoso et al., 2018). New findings show that quercetin not only has antioxidant properties but also possesses anti-inflammatory effects (Behrangi et al., 2017). It is not unexpected, as quercetin has been employed in treating numerous inflammatory disorders. Many natural antioxidants have been utilized in preclinical and clinical studies to help slow down the advancement of MS. Recent research has concentrated on the neuroprotective properties of Quercetin in the CNS for conditions like ischemia, traumatic injury, cognitive impairment, Huntington's, Parkinson's, Alzheimer's, and MS disease due to positive outcomes (Rishitha and Muthuraman, 2018). Quercetin's antioxidant abilities can hinder lipid peroxidation and lessen the production of pro-inflammatory cytokines by capturing free radicals. It should be emphasized that glycosylation can diminish the anti-inflammatory and antioxidative capabilities of Quercetin (Bentz, 2017). Quercetin (50 mg/kg) with Ethidium bromide-induced demyelination rat model was discovered to stop further demyelination, boost remyelination, enhance movement in beam walking test, hinder lipid peroxidation, and stop the reduction of acetylcholinesterase (AChE) activity (Beckmann et al., 2014). Quercetin might reduce oxidative stress and safeguard the activity of AChE in whole blood and lymphocytes as well. Different research was carried out to examine the impacts of Quercetin (25 and 50 mg/kg/day through oral gavage) on the restoration of myelin in a rat model of demyelination induced by lysolecithin (LPC) (1 %, 2 μl) in the optic chiasm (Naeimi et al., 2018). Quercetin has the ability to reduce cell death, boost cell growth, and enhance cell specialization following injury caused by lack of oxygen and glucose through activation of the PI3K/Akt signaling pathway. Ghasemi-Kasman and colleagues (2017) revealed Quercetin (50 and 100 mg/kg/day) affects the activation of astrocytes and remyelination in rats with local demyelination induced by LPC in the optic chiasm. Nanoparticles containing quercetin (25 mg/kg) promoted myelin regeneration, decreased glial activation, and inflammation (Ghasemi-Kasman et al., 2017). Quercetin possesses the capacity to attenuate oxidative stress and inflammation through the modulation of the nuclear factor erythroid 2–related factor 2 and heme oxygenase-1 signaling pathways, thereby potentially impeding the progression of neurodegenerative diseases (Javanbakht et al., 2023). Quercetin has the potential to enhance neuronal viability by promoting the expression of neurotrophic proteins that are integral to neuritic outgrowth, including growth-associated protein 43 (GAP-43), microtubule-associated protein (Moosavi et al., 2015). Quercetin can facilitate the transformation of microglial cells into the M2 phenotype, and then, M2 microglia release IL-4, which helps decrease local inflammation and neuronal damage. Furthermore, IL-10 released by M2 microglia can diminish demyelination, enhance remyelination, and result in functional enhancement (Tan et al. 2021). Quercetin was identified to stop further demyelination, enhance remyelination, boost locomotor performance in the beam walking assessment, hinder lipid peroxidation, and avert the suppression of acetylcholinesterase activity (Carvalho et al. 2018). Positive outcomes of Quercetin on the demyelination model of MS were linked to its strong interaction with cholinergic neurotransmission (Carvalho et al. 2018). Quercetin administration (10 mg/kg/day, orally) in the EAE model may reduce EAE progression by regulating myeloperoxidase activity, nitric (Mirzazadeh et al., 2019). Our findings for the first time revealed that Quercetin improves reflexive motor behavior in mice which there is no report to compare the findings with it.

Quercetin was found to improve cognitive and behavioral deficits in models of Parkinson's disease as well as in models of chronic cerebral ischemia. In addition, the administration of quercetin enhances cognitive deficits in models of learning and memory impairment induced by colchicine, scopolamine, and aluminum. Free radicals cause lipid peroxidation in biological membranes and also harm proteins and DNA as a part of the aging process (Gugliandolo et al., 2020). Quercetin treatment significantly reduced AFB1-induced oxidative stress, as demonstrated by the significant reduction of MDA marker of lipid peroxidation and by the increase in GSH, SOD, CAT levels in the brain tissue of mice exposed to AFB1 and treated with quercetin Gugliandolo et al., (2020) and our findings were in agreement to this report. Levels of some proinflammatory cytokines such as TNF-α and IL-1β in the brain play a key role in the induction of behavioral alterations, and in the induction of neuroinflammatory and therefore neurodegenerative processes (Hennessy et al., 2017; Garber et al., 2018). As observed, in the current study, CPZ administration increased TNF-α and IL-1β levels with adverse effect on the reflexive motor behavior and we think observed results might regulate via this role of the CPZ.

In conclusion, these results suggested, Quercetin has protective effect against CPZ-induced MS in mice. Based on limitations we were not able to determine histological or molecular investigations for obtained results. Further research with histological and molecular investigations suggested.

## CRediT authorship contribution statement

**Samin Ghasemi:** Data curation, Writing – original draft. **Shahin Hassanpour:** Formal analysis, Methodology, Supervision, Writing – review & editing. **Razieh Hosseini:** Project administration.

## Declaration of competing interest

No conflict of interest.

## Data Availability

No data was used for the research described in the article.
